# Comparing the reliability and validity of the SF-36 and SF-12 in measuring quality of life among adolescents in China: a large sample cross-sectional study

**DOI:** 10.1186/s12955-020-01605-8

**Published:** 2020-11-09

**Authors:** Yanwei Lin, Yulan Yu, Jiayong Zeng, Xudong Zhao, Chonghua Wan

**Affiliations:** 1 Department of Health Sociology, School of Humanities and Management, Guangdong Medical University, 1#, Xincheng Avenue, Songshanhu District, Dongguan, 523808 Guangdong China; 2 Department of Psychology, School of Humanities and Management, Guangdong Medical University, 1#, Xincheng Avenue, Songshanhu District, Dongguan, 523808 Guangdong China; 3 Institute of Psychosomatic Medicine, the East Translational Medicine Platform of Tongji University, 50#, Chifeng Avenue, Shanghai, 200092 China; 4 Research Center for Quality of Life and Applied Psychology, Key Laboratory for Quality of Life and Psychological Assessment and Intervention, Guangdong Medical University, 1#, Xincheng Avenue, Songshanhu District, Dongguan, 523808 Guangdong China

**Keywords:** Quality of life, Reliability, Validity, Discrimination, Average information, SF36, SF12, Chinese adolescents

## Abstract

**Objective:**

We compare the reliability and validity of the Short Form 36 (version 1, SF-36) and the Short Form 12 (version 1, SF-12) in adolescence, the period of life when a child develops into an adult, i.e., the period from puberty to maturity terminating legally at the age of majority (10–19 years), thus supplying evidence for the selection of instruments measuring the quality of life (QOL) and decision-making processes of adolescents in China.

**Methods:**

Stratified cluster random sampling was adopted according to geographical location, and the SF-36 was administered to assess QOL. The Pearson correlation coefficient was used to show correlation. Cronbach’s alpha and construct reliability (CR) were used to evaluate the reliability of SF-36 and SF-12, while criterion validity and average variance extracted (AVE, convergence validity) were used to evaluate validity. Confirmatory factor analysis was used to calculate the load factors for the items of the SF-36 and SF-12, then to obtain the CR and AVE. The Semejima grade response model (logistic two-parameter module) in item response theory was used to estimate item discrimination, item difficulty, and item average information for the items of the SF-36 and SF-12.

**Results:**

19,428 samples were included in the study. The mean age of respondents was 14.78 years (SD = 1.77). Reliability of each domain of the SF-36 was better than for the corresponding domain of the SF-12. The domains of PF, RP, BP, and GH in SF-36 had good construct reliability (CR > 0.6). The criterion validities of some domains of the SF-36 were a little higher in some corresponding dimensions of the SF-12, except for PCS. The convergence validities of the SF-12 were higher than the SF-36 in PF, RP, BP, and PCS. The items of BP, SF, RP, and VT in the SF-12 had acceptable discrimination of items that were higher than in the SF-36. The items’ average amounts of information on BP, VT, SF, RE, and MH in the SF-36 and SF-12 were poor.

**Conclusion:**

Two component (PCS and MCS) measurements of the SF-12 appeared to perform at least as well as the SF-36 in cross-sectional settings in adolescence, but the reliability and validity of the 8 domains of the SF-36 were better than those of the SF-12. Some domains, for instance SF and BP, were not suitable for adolescents or need to be studied further.

## Introduction

Youth involves identity-building. Experiences during this developmental period can shape long-term attributes and attitudes and may lead to the adoption of a lifetime of healthy or risky behaviors [1]. The determinants of current and future health and disease for adolescents span the social and psychological fields [2]. A deeper understanding of how adolescents view their lives allows a greater understanding of their present health. The health-related quality of life (HRQOL) of school-age adolescents has been the subject of international interest. The term refers to a comprehensive model of subjective health that covers physical, social, psychological, and functional aspects of individual well-being as a multidimensional and subjective construct [3, 4]. The point of all this interest is to guide the organization of resources and decision-making processes to promote adolescents’ quality of life. To accomplish this, understanding the current quality of adolescents’ life is essential [5, 6].

The SF-36 was developed and validated as a generic short-form instrument for measuring HRQOL; it was widely applied to assess important QOL domains in the Medical Outcomes Study [7]. The SF-36 consists of eight QOL domains: PF, physical functioning; RP, role physical; BP, bodily pain; GH, general health; VT, vitality; SF, social functioning; RE, role emotional; and MH, mental health; with two summary components having been constructed to summarize the physical and mental components (PCS and MCS, respectively) [8]. The factor structures of SF-36 that have been identified in China suggest that PCS is primarily a comprehensive measure of PF, RP, BP, and GH and that MCS mainly encompasses the domains of VT, SF, RE, and MH. However, the two components somewhat overlap, and especially the VT, GH, and SF domains have noteworthy correlations with both components [9].

One of the major advantages of using the SF-36 is that it allows for QOL scores to be compared with scores in different groups [10]. However, because the SF-36 was not originally designed to measure important aspects of the QOL of adolescents specifically, some studies have determined that the instrument, especially the mental component (MCS), is relatively insensitive to variations in different populations over time [11–13].

A substantially shorter instrument, the SF-12 was developed by Ware and colleagues, reducing the number of items from 36 to 12 to create an abbreviated version of the SF-36.[14, 15]. Most of the respondents testing the new instrument completed the SF-12 in less than a third of the usual time needed to complete the SF-36 [8]. Ware showed that the two instruments are highly correlated, and about 90% of the variation in both the physical and the mental component summaries measured in the SF-36 was explained by the same summary measures of the SF-12 [16]. Subsequent studies comparing the two scales have suggested varying results based upon the disease or health condition of interest [17–19]. The SF-12 and SF-36 are available in many languages and have been applied to all kinds of groups, including adolescents [15, 20–22]. Since studies have demonstrated that both scales are valid instruments for this age group, they have been used to evaluate the QOL of adolescents in China [23] as more and more studies have focused on the quality of life of healthy adolescents in that country.

Most studies of adolescent QOL in China have surveyed the perception of QOL among chronically ill adolescent patients and were conducted in hospital or outpatient settings [25, 26]. Recently there has been a growing interest in the study of healthy groups of adolescents, leading to studies being performed in other contexts, such as in schools [27, 28], because of a growing awareness of the need to recognize and monitor adolescents who are most vulnerable to a poor health-related quality of life [29, 30]. In some studies, though the SF-12 and SF-36 were used to investigate perceived adolescent QOL, it was unclear which of the two instruments was more suitable to the age group [23]. Thus, our study aimed to evaluate the QOL of healthy adolescent students at schools in China by using the SF-36 and SF-12 and comparing the reliabilities and validities of both, supplying evidence for the selection of instruments measuring quality of life and decision-making processes and thereby promoting the quality of life of adolescents.

## Methods

### Study design and sample

Stratified cluster random sampling was adopted [31], first dividing regions by geographical location: Dongguan, Shanghai, Shenyang, Wuhan, Xi’an, and Kunming represented the south, east, north, central, northwest, and southwest regions, respectively. These areas were chosen in order to ensure proper representation by including participants from geographically diverse areas. Second, middle schools were randomly selected and followed by grade (first grade of junior school to third grade of high school). The basic sampling frameworks were all middle schools, as reported by each city. In each city, middle schools were selected by simple random sampling according to a random number table. Finally, 17 middle schools were included (4 in Dongguan, 1 in Shanghai, 3 in Shenyang, 1 in Wuhan, 4 in Xi’an, and 4 in Kunming). The number of schools in each city was limited by the research group's local investigative capacity.

All students enrolled from the first grade of junior high school to the third grade of high school were included in the survey. The exclusion criteria were those with any physical or mental condition that made them unable to complete questionnaires or students and their parents who had not signed an informed consent form. The study was approved by the Institutional Review Board (IRB) at the Affiliated Hospital of Guangdong Medical University. Verbal informed consent for publication was obtained from the participants and/or their relatives, as approved by the IRB. The response rate was almost 80%. This present study included 19,428 adolescents with complete information on quality of life measures. The sample sizes for each region were Dongguan (4490, 23.1%), Shanghai (1039, 5.3%), Shenyang (3539, 18.2%), Wuhan (1371, 7.1%), Xi’an (4197, 21.6%), and Kunming (4792, 24.7%).

### Instruments and variables

SF-36 (version 1) was used to assess QOL. Compared with version 2, the differences lie in two points. First, the answers-rank of RP, RE, MH, and VT are distinct, and second, the scoring rules are different [32]. Since the use of SF-36 (version 2) requires authorization, version 1 was used in this study. Based on the response to individual items comprising the 8 subscales and using a z-score transformation, the scores of each subscale were calculated [33]. First, the domain items were coded; second, the items were scored; and finally, the scores were converted as shown in Formula 1.

1 $${\text{Score }} = \frac{actual\; score - the \;lowest\; possible\; score \;of \;the\; subscale}{{the\; highest\; score\; of\; the\; subscale - the\; lowest\; score\; of\; the\; subscale}} \times 100\%$$

Scoring norms for the Chinese version of the SF-36 (version 1) and SF-12 are not given at present by studies, so scores of these instruments were mainly based on American norms in China that have been proven to be valid [23, 32, 34]. Using Z-transform scores and factor score coefficients, we calculated PCS and MCS scores of the SF-36 according to Formulas 2 and 3:

2 $${\text{PCS}}\_{\text{T }} = 50 + 0.424{\text{PF}} + 0.351{\text{RP}} + 0.318{\text{BP}} + 0.250{\text{GH}} + 0.029{\text{VT}} + \left( { - 0.008} \right){\text{SF}} + \left( { - 0.192} \right){\text{RE}} + \left( { - 0.221} \right){\text{MH}}$$

3 $${\text{MCS}}\_{\text{T}} = 50 + \left( { - 0.230} \right){\text{PF}} + \left( { - 0.123} \right){\text{RP}} + \left( { - 0.097} \right){\text{BP}} + \left( { - 0.016} \right){\text{GH}} + 0.235{\text{VT}} + 0.268{\text{SF}} + 0.434{\text{RE}} + 0.486{\text{MH}}$$

SF-12 component summary scores (eight subscales, PCS-12, and MCS-12) were calculated using the SF-12 items that are embedded in the SF-36 [35]. This method has been presented as being equivalent to calculating the SF-12 as a stand-alone questionnaire [17]. All summary scores range from 0 to 100, where higher scores indicate better QOL. We calculated PCS and MCS scores of the SF-12 according to the SF-12 scoring algorithm proposed by John E Ware in 1995 that has been widely used in China [36].

### Statistical analysis

For descriptive analyses, we aimed to show overall demographics and QOL. We calculated average and standard deviations in QOL scores by SF-36 and SF-12. For testing their relevance, the Pearson correlation coefficient was used to show correlation between the domains of SF-36 and SF-12.

Cronbach’s alpha for domains composed of multiple items and construct reliability (CR) were used to evaluate the reliability of the SF-36 and the SF-12, and validity indicators were represented by criterion validity and convergence validity (average variance extracted, AVE). Criterion validity was expressed by the correlation between the response of each domain and self-reported health status. The calculation of the formulas for CR and AVE are shown in Formulas 4 and 5.

4 $$CR = \frac{{(\sum \lambda )^{2} }}{{(\sum \lambda )^{2} + \sum \theta }}$$

5 $${\text{AVE}} = \frac{{\left( {\sum {\lambda ^{2} } } \right)}}{{\left( {\sum {\lambda ^{2} } } \right) + \sum \theta }}$$

λ = factor loading, θ = measurement error.

The sample was randomly split into a training set (50%) and a validation set (50%) to examine the construct validity of the SF-36 and SF-12. Using the training set, an exploratory factor analysis (EFA) was performed to explore the latent structure based on correlations matrix, and factor loadings (λ) were estimated by maximum likelihood estimation and rotated by promax. Using the validation set, confirmatory factor analysis (CFA) was used to validate the identified two-factor structure in some Chinese populations. The weighted least-square method was used for the estimation of CFA parameters. Factor loadings (λ) were taken for standardized regression coefficients. The classic goodness-of-fit χ^2^ statistic and its degrees of freedom were reported. However, as the χ^2^ statistic is highly sensitive in large samples, assessment of goodness-of-fit was based on the fit indices as recommended: the root mean square error of approximation (RMSEA, close to 0.06 or lower) and the comparative fit index (CFI, close to 0.95) [37]. The basic two-factor CFA model (Model I) without correlated errors was first assessed (PCS associated with PF, RP, BP, and GH, whereas MCS associated with VT, SF, RE, and MH). Subsequently, the factor structures PCS and MCS, associated with most of the 8 domains (Model II) as described above, were also incorporated [23]. Then, the EFA or CFA was repeated on another data set, and mean estimates were reported.

According to the evaluation results of the samples, and taking into account the characteristics of the ordered and multi-category forms of the instrument items, the Semejima grade response model (logistic two-parameter module) in item response theory was used to estimate the discrimination, difficulty, and average information of each item [38]. RStudio, Amos 20.0, and Multilog 7.03 were used to process data.

## Results

### Sample characteristics

Of the 20,226 questionnaires received, 798 had no responses on some of the SF-36 items. In the end, 19,428 samples were included in the study. The mean age of the sample of respondents was 14.78 years (standard deviation [SD] = 1.77), and 49.4% (9,595) were boys. Among the SF-36 and SF-12 domains, the PF mean score was the highest, and the RE mean score was the lowest. PCS was better than MCS. The biggest mean difference in scores between the two instruments was in the domain of SF. Of the corresponding domains, the RE domains were the most relevant (r = 0.923), while the smallest correlation coefficient was in the VT domains (r = 0.670), which means domains of the SF-12 could reflect the information from 67.0% to 92.3% of the corresponding domains of the SF-36 (Table 1).

**Table 1:** Scores of SF-36 and SF-12 among adolescents (n = 19,428)

	SF-36	SF-12	Mean difference	Correlation coefficient
PF***	89.10 ± 14.39	91.64 ± 16.85	− 2.54	0.800
RP***	68.86 ± 34.28	68.08 ± 39.44	0.78	0.897
BP***	79.97 ± 19.77	85.09 ± 19.25	− 5.12	0.876
GH***	70.41 ± 19.53	62.72 ± 26.39	7.69	0.670
VT***	65.04 ± 17.19	62.11 ± 25.90	2.93	0.645
SF***	77.98 ± 19.07	66.17 ± 23.17	11.81	0.875
RE***	54.82 ± 37.45	52.14 ± 40.44	2.68	0.923
MH***	68.51 ± 17.18	64.86 ± 18.83	3.65	0.799
PCS***	75.00 ± 11.10	70.52 ± 13.65	4.48	0.812
MCS***	68.55 ± 14.18	61.32 ± 7.17	7.23	0.779

*PF* physical functioning, *RP* role physical, *BP* bodily pain, *GH* general health, *VT* vitality, *SF* social functioning, *RE* role emotional, *MH* mental health, *PCS* physical component summary, *MCS* mental component summary

^*^p < 0.05; **p < 0.01; ***p < 0.00

### The reliability and validity in classical test theory

#### Factor analysis by EFA

The construct validity of SF-36 was good in adolescents, as determined by the Kaiser–Meyer–Olkin Measure of Sampling Adequacy (0.884). Communalities of all of variables were over 0.5. Factors rotated by the varimax method such that eigenvalues were greater than 1 were extracted. Eight components were produced and explained 69.21% of the total variance. The structure loading of factors extracted and the component score coefficient matrix are presented in Table 2. The structure of the 8 domains identified (PF, RP, BP, GH, VT, SF, RE, and MH) was not supported by EFA. The domains of BP, SF, VT, and MH were not divided into identified structures, due to the strong correlations between BP and SF and between VT and MH. Details are shown in Table 2.

**Table 2:** Results of factors analysis of SF-36 among adolescents (n = 9741)

	1-PF	2-PF	3-RP	4-BP\SF	5-GH	6-SF\VT\MH	7-MH\VT	8-RE
PF01	–	0.794	–	–	–	–	–	–
PF02	–	0.668	–	–	–	–	–	–
PF03	–	0.600	–	–	–	–	–	–
PF04	0.708	–	–	–	–	–	–	–
PF05	0.844	–	–	–	–	–	–	–
PF06	0.631	–	–	–	–	–	–	–
PF07	0.342	–	–	–	–	–	–	–
PF08	0.618	–	–	–	–	–	–	–
PF09	0.593	–	–	–	–	–	–	–
PF10	0.694	–	–	–	–	–	–	–
RP1	–	–	0.689	–	–	–	–	–
RP2	–	–	0.709	–	–	–	–	–
RP3	–	–	0.726	–	–	–	–	–
RP4	–	–	0.692	–	–	–	–	–
BP1	–	–	–	0.766	–	–	–	–
BP2	–	–	–	0.774	–	–	–	–
GH1	–	–	–	–	0.625	–	–	–
GH2	–	–	–	–	0.654	–	–	–
GH3	–	–	–	–	0.723	–	–	–
GH4	–	–	–	–	0.577	–	–	–
GH5	–	–	–	–	0.751	–	–	–
VT1	–	–	–	–	–	–	0.775	–
VT2	–	–	–	–	–	–	0.660	–
VT3	–	–	–	–	–	0.701	–	–
VT4	–	–	–	–	–	0.746	–	–
SF1	–	–	–	0.570	–	–	–	–
SF2	–	–	–	–	–	0.555	–	–
RE1	–	–	–	–	–	–	–	0.690
RE2	–	–	–	–	–	–	–	0.725
RE3	–	–	–	–	–	–	–	0.688
MH1	–	–	–	–	–	0.660	–	–
MH2	–	–	–	-	–	0.783	–	–
MH3	–	–	–	–	–	–	0.710	–
MH4	–	–	–	–	–	0.731	–	–
MH5	–	–	–	–	–	–	0.706	–

Similarly, the construct validity of the SF-12 was also good in adolescents; the Kaiser–Meyer–Olkin Measure of Sampling Adequacy was 0.732. Eight components were extracted and explained 63.50% of the total variance. Due to the strong correlations between MH and SF and between VT and MH, the domains of SF, VT, and MH were not divided into identified structures in the SF-12 (Table 3).

**Table 3:** Results of factors analysis of SF-12 among adolescents (n = 9741)

	1-PF	2-RP	3-BP	4-GH	5-VT/MH	6-SF\MH	7-RE	8-RE
PF02	0.808	–	–	–	–	–	–	–
PF04	0.829	–	–	–	–	–	–	–
RP2	–	0.742	–	–	–	–	–	–
RP3	–	0.872	–	–	–	–	–	–
BP2	–	–	0.951	–	–	–	–	–
GH1	–	–	–	0.949	–	–	–	–
VT2	–	–	–	–	0.696	–	–	–
SF2	–	–	–	–	–	0.766	–	–
RE2	–	–	–	–	–	–	0.865	–
RE3	–	–	–	–	–	–	–	0.929
MH3	–	–	–	–	0.872	–	–	–
MH4	–	–	–	–	–	0.855	–	–

#### Factor analysis by CFA

**Fig. 1:**
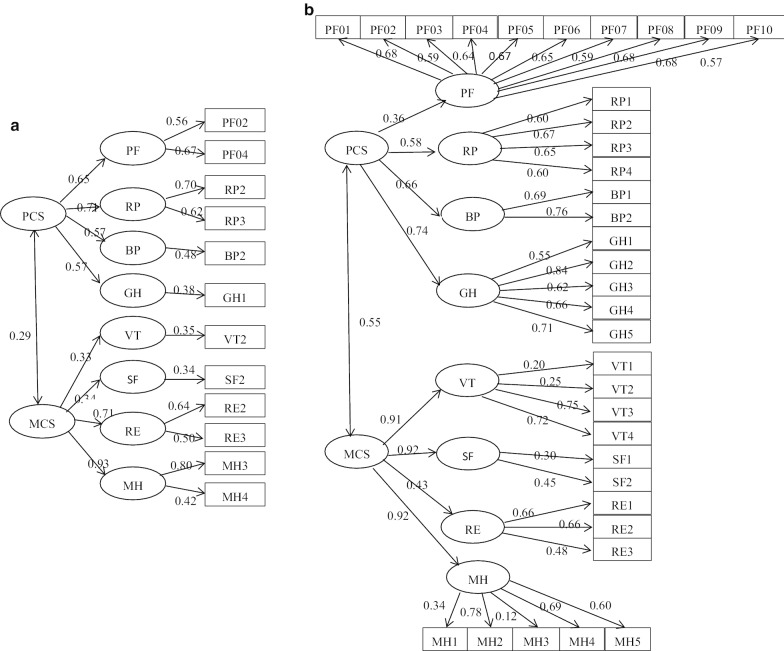
Standardized parameter estimates for confirmatory factor analysis of the Short Form-12(a) and the Short Form-36(b)

We confirmed two conceptual models. Conceptual Model I assumed that PCS was associated with PF, RP, BP, and GH, whereas MCS was associated with VT, SF, RE, and MH. Conceptual Model II assumed that PCS and MCS were associated with most of the 8 domains. Fit indices of the two models revealed that no matter whether SF-36 or SF-12, Conceptual Model I was better than Conceptual Model II in the structures identified (Table 4). The structure of Model I has been used widely in studies in China. In our study, we selected the structures of Model I as the two summary scales (PCS and MCS) of the SF-36 and the SF-12. Standardized parameter estimates for CFA on each path are shown in Fig. 1.

**Table 4:** Two summary scales confirmed by CFA in SF-36 and SF-12 among adolescents

	SF-36						SF-12			
	Conceptual Model I			Conceptual Model II			Conceptual Model I		Conceptual Model II	
	PCS		MCS	PCS		MCS	PCS	MCS	PCS	MCS
PF	0.363		–	0.244		0.394	0.652	–	0.559	0.179
RP	0.583		–	0.358		0.465	0.705	–	0.778	0.144
BP	0.663		–	0.656		0.194	0.572	–	0.697	0.231
GH	0.737		–	0.758		0.247	0.566	–	0.564	0.243
VT	–		0.909	0.357		0.839	–	0.334	0.158	0.579
SF	–		1.119	0.470		1.041	–	0.342	0.102	0.645
RE	–		0.429	0.280		0.406	–	0.707	0.210	0.748
MH	–		0.915	0.450		0.726	–	0.932	0.098	0.892
*Fit indices for 2-factor confirmatory factor analysis (n* = *9741)*										
χ^2^ statistic (df)		6948.000 (551)			20,771.000 (551)		3089.478 (49)		5769.000 (49)	
RMSEA (90% CI)		0.061 (0.060, 0.063)			0.075 (0.074, 0.075)		0.060 (0.058, 0.062)		0.080 (0.078, 0.082)	
CFI		0.94			0.70		0.969		0.769

#### Validity and reliability of domains of SF-36 and SF-12

As mentioned above, standardized parameter estimates for CFA in Model I were selected as factor loading. CR and AVE were calculated according to Formulas 4 and 5.

Except for SF domains in the SF-36 (Cronbach’s α = 0.211), domains composed of multiple items had generally acceptable internal reliability (Table 2). The low internal reliability of SF domains was probably because of inconsistent understanding of the meaning of the only two items, which might be biased or difficult to parse for adolescents (“To what extent has your physical health or emotional problems interfered with…” and “How much of the time has your physical health or emotional problems interfered with…”). Moreover, consistent with related studies, the internal reliability of the MH domain in the SF-12 was low (Cronbach’s α = 0.369). On the other hand, the internal reliability of the SF-36 in each domain was better than that of the corresponding domains of the SF-12, which was consistent with higher internal reliability due to there being more items. The domains of PF, RP, BP, GH, and PCS in the SF-36 had good construct reliability (CR > 0.6). Except for RP and PCS, the domains in the SF-12 were not good at construct reliability, especially for the domains of GH, VT, and SF.

The criterion validity was calculated based on the item of self-reported health (“In general, would you say your health is….”). It is worth noting that criterion validities of all the domains of the two instruments were low, but especially so for PF, RP, and SF, which suggests that the correlation between physical health and self-perceived health was weak. Moreover, in PCS, the criterion validity of the SF-12 was much higher than the criterion validity of the SF-36. Although the criterion validities of the SF-36 were higher in other corresponding dimensions, the gaps were small.

PF, RP, and PCS had generally acceptable convergence validity whether in the SF-36 or the SF-12. Moreover, in the RP and PCS domains, the convergence validities of the SF-12 were higher than the SF-36, while there was a little bit of difference in the other domains except BP, GH, and VT (Table 5).

**Table 5:** Validity and reliability of SF-36 and SF-12 in classical test theory

	SF-36				SF-12				Difference (SF-36–SF-12)			
	Reliability		Validity		Reliability		Validity		Reliability		Validity	
	Cronbach’s alpha	CR	Criterion validity	AVE	Cronbach’s alpha	CR	Criterion validity	AVE	Cronbach’s alpha	CR	Criterion validity	AVE
PF	0.841	0.858	0.085	0.380	0.564	0.540	0.055	0.371	0.277	0.318	0.030	0.009
RP	0.727	0.728	0.173	0.405	0.605	0.602	0.171	0.432	0.122	0.126	0.002	− 0.027
BP	0.670	0.690	0.283	0.528	–	0.222	0.227	0.222	–	0.468	0.056	0.306
GH	0.766	0.781	0.670	0.420	–	0.134	–	0.134	–	0.647	–	0.286
VT	0.569	0.577	0.309	0.302	–	0.117	0.252	0.117	–	0.460	0.057	0.185
SF	0.211	0.329	0.113	0.146	–	0.112	0.027	0.112	–	0.217	0.086	0.034
RE	0.626	0.489	0.203	0.371	0.485	0.491	0.199	0.329	0.141	– 0.002	0.004	0.042
MH	0.625	0.426	0.243	0.316	0.396	0.360	0.049	0.303	0.229	0.066	0.194	0.013
PCS	0.562	0.935	0.350	0.430	0.422	0.719	0.589	0.392	0.14	0.216	− 0.239	0.038
MCS	0.609	0.418	0.476	0.299	0.429	0.690	0.300	0.399	0.18	–0.272	0.176	− 0.1

Construct reliability = CR, average variance extracted = AVE = convergence validity

### Validity and reliability in item response theory

The parameter values and information content of the items according to the Samezima grade response model are shown in Table 6. The discriminations of items were between 0.45–2.73, with a large gap. The difficulty of the items ascended from the lowest level to the highest level unidirectionally, which met the difficulty assumptions estimated by the model. The average amount of information of each item was between 0.07 and 1.02.

**Table 6:** Item discrimination, difficulty, and average amount of information in item response theory

Label	SF-36			SF-12		
	Item discrimination (SD)	Item difficulty (SD)	Average amount of information	Item discrimination (SD)	Item difficulty (SD)	Average amount of information
*Physical functioning (PF)*						
PF01	2.73 (0.01)	− 1.43 (0.01)	1.02			
		0.21 (0.01)				
PF02	2.73 (0.01)	− 2.53 (0.05)	0.74	2.20 (0.03)	− 3.13 (0.05)	0.45
		− 1.07 (0.01)			− 1.40 (0.02)	
PF03	2.73 (0.01)	− 2.55 (0.05)	0.73			
		− 1.14 (0.01)				
PF04	2.73 (0.01)	− 2.05 (0.03)	0.9	2.20 (0.03)	− 2.60 (0.04)	0.54
		− 0.87 (0.01)			− 1.17 (0.01)	
PF05	2.73 (0.01)	− 2.45 (0.04)	0.65			
		− 1.54 (0.02)				
PF06	2.73 (0.01)	− 1.88 (0.02)	0.89			
		− 0.90 (0.01)				
PF07	2.73 (0.01)	− 1.42 (0.01)	0.95			
		− 0.25 (0.01)				
PF08	2.73 (0.01)	− 1.96 (0.03)	0.89			
		− 0.92 (0.01)				
PF09	2.73 (0.01)	− 2.51 (0.05)	0.63			
		− 1.58 (0.02)				
PF10	2.73 (0.01)	− 1.69 (0.02)	0.74			
		− 1.20 (0.01)				
*Role physical (RP)*						
RP1	2.17 (0.02)	0.77 (0.01)	0.43			
RP2	2.17 (0.02)	0.53 (0.01)	0.43	2.32 (0.03)	0.52 (0.01)	0.46
RP3	2.17 (0.02)	0.65 (0.01)	0.43	2.32 (0.03)	0.63 (0.01)	0.46
RP4	2.17 (0.02)	0.52 (0.01)	0.43			
*Bodily pain (BP)*						
BP1	0.45 (0.01)	− 10.26 (0.48)	0.06			
		− 8.08 (0.31)				
		− 4.60 (0.16)				
		− 1.33 (0.06)				
		1.24 (0.06)				
BP2	0.45 (0.01)	0.32 (0.05)	0.05	1.06 (0.02)	0.18 (0.02)	0.24
		4.65 (0.17)			2.40 (0.04)	
		7.46 (0.28)			3.83 (0.07)	
		10.00 (0.44)			5.28 (0.12)	
*General health (GH)*						
GH1	1.76 (0.01)	− 3.05 (0.05)	0.76	0.91 (0.01)	− 1.80 (0.03)	0.24
		− 1.11 (0.02)			0.21 (0.02)	
		− 0.13 (0.01)			1.72 (0.03)	
		1.2 (0.01)			4.93 (0.09)	
GH2	1.76 (0.01)	− 2.33 (0.03)	0.73			
		− 1.46 (0.02)				
		− 0.23 (0.01)				
		0.54 (0.01)				
GH3	1.76 (0.01)	− 2.77 (0.03)	0.68			
		− 2.14 (0.02)				
		− 0.89 (0.01)				
		0.35 (0.01)				
GH4	1.76 (0.01)	− 2.43 (0.03)	0.67			
		− 1.55 (0.02)				
		− 0.52 (0.01)				
		0.17 (0.01)				
GH5	1.76 (0.01)	− 2.75 (0.03)	0.71			
		− 2.04 (0.02)				
		− 0.78 (0.01)				
		0.57 (0.01)				
*Vitality (VT)*						
VT1	0.74 (0.00)	− 2.43 (0.04)	0.17			
		0.29 (0.03)				
		1.68 (0.03)				
		3.33 (0.05)				
		4.71 (0.07)				
VT2	0.74 (0.00)	− 2.74 (0.04)	0.17	0.91 (0.01)	− 2.36 (0.01)	0.25
		− 0.40 (0.03)			− 0.35 (0.02)	
		1.22 (0.03)			1.07 (0.02)	
		2.89 (0.04)			2.50 (0.04)	
					3.90 (0.07)	
VT3	0.74 (0.00)	− 4.73 (0.07)	0.16			
		− 3.10 (0.04)				
		− 1.97 (0.03)				
		− 0.50 (0.03)				
		2.10 (0.03)				
VT4	0.74 (0.00)	− 4.26 (0.06)	0.18			
		− 2.55 (0.04)				
		− 1.36 (0.03)				
		0.15 (0.03)				
		2.93 (0.04)				
*Social functioning (SF)*						
SF1	0.50 (0.01)	− 1.68 (0.06)	0.07			
		2.80 (0.08)				
		5.92 (0.17)				
		8.66 (0.29)				
SF2	0.50 (0.01)	− 6.35 (0.18)	0.07	1.07 (0.02)	− 3.42 (0.06)	0.28
		− 4.73 (0.13)			− 2.58 (0.04)	
		− 3.48 (0.10)			− 1.92 (0.03)	
		− 2.06 (0.07)			− 1.15 (0.02)	
		− 0.01 (0.05)			− 0.02 (0.02)	
*Role emotional (RE)*						
RE1	1.82 (0.02)	0.35 (0.01)	0.36			
RE2	1.82 (0.02)	0.23 (0.01)	0.36	1.63 (0.02)	0.24 (0.01)	0.31
RE3	1.82 (0.02)	− 0.07 (0.01)	0.36	1.63 (0.02)	− 0.07 (0.01)	0.32
*Mental health (MH)*						
MH1	0.78 (0.00)	− 4.35 (0.07)	0.19			
		− 2.59 (0.04)				
		− 1.53 (0.03)				
		− 0.33 (0.03)				
		1.50 (0.03)				
MH2	0.78 (0.00)	− 4.49 (0.07)	0.18			
		− 2.99 (0.04)				
		− 2.12 (0.03)				
		− 1.01 (0.03)				
		0.82 (0.03)				
MH3	0.78 (0.00)	− 11 (–)	0.19	0.79 (0.01)	− 3.94 (0.06)	0.2
		− 2.84 (0.07)			− 2.24 (0.03)	
		− 0.42 (0.03)			− 0.82 (0.03)	
		1.03 (0.03)			0.55 (0.03)	
		2.72 (0.04)			2.91 (0.04)	
MH4	0.78 (0.00)	− 4.83 (0.08)	0.18	0.79 (0.01)	− 4.73 (0.07)	0.18
		− 3.18 (0.05)			− 3.18 (0.04)	
		− 2.15 (0.03)			− 2.19 (0.03)	
		− 0.82 (0.03)			− 0.88 (0.03)	
		2.04 (0.03)			2.00 (0.03)	
MH5	0.78 (0.00)	− 11.18 (–)	0.18			
		− 1.35 (0.04)				
		0.84 (0.03)				
		2.05 (0.03)				
		3.48 (0.05)				
HT	0.91 (0.00)	− 4.84 (0.09)	0.23			
		− 2.59 (0.04)				
		− 0.23 (0.02)				
		1.25 (0.03)				

*SD* standard deviation

In the SF-36, the domains of PF, RP, GH, and RE had acceptable discrimination of items (> 1), but the remaining dimensions were less differentiated, especially BP and SF, probably because for teenagers there was strong homogeneity between individuals in terms of physical pain and social function. On the other hand, in the SF-12, BP, SF, RP, and VT had higher discrimination of items than in the SF-36.

With reference to the relevant literature, the amount of information measured on the scales > 25 indicated that the quality of the evaluation items was good; the amount of information < 16 indicates that the evaluation items were poor [31]. Given the number of items on the instrument for the SF-36, we divided 25 and 16 by 36 to get the average information amount for each item, so as to obtain the determination criterion: the average information amount of excellent items was > 0.69 (25/36), while items < 0.44 (16/36) were judged to be poor. Similarly, for the SF-12, the average information amount of the excellent items was > 2.08, while items < 1.33 were judged to be poor. Except for PF05 and PF09, the items of the PF domain in the SF-36 were excellent, and the items of the GH domain in the SF-36 were excellent too, though the items of BP, VT, SF, RE, and MH were poor. On the other hand, the average amounts of information in the SF-12 items were poor (Table 6).

## Discussion

Psychometric standards were used to evaluate the reliability and validity of the standard Chinese SF-36 and SF-12 instruments in a large sample of Chinese adolescents. Our study suggested that the SF-12 and the SF-36 correlated very highly in this population. Although the reliability and average amount of information of the SF-12 domains and items were lower than that of the SF-36, the convergence validity and item discrimination of some domains in the SF-12 were somewhat better than the corresponding domains in the SF-36. No matter whether the SF-36 or the SF-12 was considered, high correlations existed between some domains, for example, between MH and VT, SF dimensions, etc. The psychometric properties of the two broader components (PCS and MCS) were better than the individual domains.

Studies have shown that the two instruments discriminated between adolescents with physical and mental health problems and performed well in associating with other clinical criteria [39–41]. A study of 31,357 adolescents in Hong Kong showed the two components and a single general health component of the standard Chinese SF-12 were appropriate health indicators for Chinese adolescents [23]. Studies have also shown that the SF-12 correlated highly with the SF-36 in obese and non-obese patients [3, 4]. However, many problems with the two instruments still existed, such as a high correlation between the two components, low internal reliability, and the ceiling effect within individual domains [42]. Comparing the SF-12 and the SF-36, previous studies in patients with specific diseases or health conditions have generally found moderate to high correlations between corresponding domains and components of both instruments [15, 19]. Our study also demonstrated these correlations. Since the SF-12 is embedded in the SF-36, we expected reasonably high correlations. Overall, the dimensions of the SF-12 scale could reflect 64.5% to 92.3% of the corresponding dimensions of the SF-36 scale in Chinese adolescents, with low internal reliabilities and convergence validities found in some domains.

A low reliability and validity of the social functioning domain was noted. This might indicate questionable reliability and validity of the instruments or the lack of representation [3]. On the other hand, it might also be attributed to the presence of inconsistent responses, which might occur if respondents completed a questionnaire without comprehending the items, as might occur with adolescents [23]. Due to the brevity of the SF-12 instrument, related research has shown that it is not possible to get reliable information for each of the eight domains, so that one would not be able to draw conclusions about specific domains [43]. Indeed, we found the SF-36 was better than the SF-12 in terms of reliability. At the same time, comparing the SF-12 and the SF-36 in terms of validity, no loss in effectiveness was shown, and there was even a slight improvement. But we also found that the criterion validities of PF, SF, and MH were low. Relevant literature has found that for most adolescents, performing moderately strenuous activities or climbing several flights of stairs would not present problems because this age group is typically physically fit and active, but when combined with a limited social life and less satisfactory mental state, inconsistent responses would be possible [23].

Unlike previous studies [21, 42–44], we found the domains of BP and SF in general had poor discrimination of items, while PF in general, as well as BP, SF, RP, and VT on the SF-12, had higher discrimination of items than in the SF-36. We suggest that, compared with PF items, the items in these other domains were not easy for teenagers to understand, resulting in a lack of sensitivity in the measurement. Similarly, a loss of information had been found in the SF-12 that would be provided by the eight dimensions of the SF-36, but utilization of the two summary dimensions of the SF-12 had an advantage for adolescents, which was consistent with the results of other population studies [23].

Methodological limitations should be mentioned. The participants were stratified regarding geographical areas in order to minimize the risk of possible regional differences. However, the regions chosen were vast and included small towns and big cities as well as rural areas [45, 46]. Differences due to these circumstances might exist but not have come to light in this design. Additionally, there was a difference in response consistency between the samples because of the characteristics of adolescence, leading to bias in the results [47].

## Conclusion

In general, our study suggested that the SF-12 correlated highly with the SF-36 in adolescent groups in China. If focus is restricted to the two broad component measurements (PCS and MCS), the SF-12 appeared to perform at least as well as the SF-36 in cross-sectional settings in adolescence; hence, using the SF-12 in place of the SF-36 might be appropriate in this situation. At the same time, the question of whether some domains, for instance SF and BP, are not suitable for adolescents needs further study.

## Data Availability

The study data is available upon request.

## Abbreviations

QOL: Quality of life
SF-36: The Short-Form 36
SF-12: The Short-Form 12
PF: Physical functioning
RP: Role physical
BP: Bodily pain
GH: General health
VT: Vitality
SF: Social functioning
RE: Role emotional
MH: Mental health
PCS: Physical component summary
MCS: Mental component summary
CR: Construct reliability
AVE: Average variance extracted
